# Evaluation of Antimicrobial Therapy of Blood Culture Positive Healthcare-Associated Infections in Children

**DOI:** 10.1371/journal.pone.0141555

**Published:** 2015-11-05

**Authors:** Niina Laine, Martti Vaara, Veli-Jukka Anttila, Kalle Hoppu, Raisa Laaksonen, Marja Airaksinen, Harri Saxen

**Affiliations:** 1 Children´s Hospital, Helsinki University Central Hospital (HUCH), Helsinki, Finland; 2 Division of Pharmacology and Pharmacotherapy, Faculty of Pharmacy, University of Helsinki, Helsinki, Finland; 3 Division of Clinical Microbiology, HUSLAB, Helsinki University Central (HUCH) Hospital, Helsinki, Finland; 4 Department of Infectious Diseases, Helsinki University Central Hospital (HUCH), Helsinki, Finland; 5 Poison Information Centre, Helsinki University Central Hospital (HUCH), Helsinki, Finland; Ross University School of Veterinary Medicine, SAINT KITTS AND NEVIS

## Abstract

**Aim:**

Knowledge of the quality of antimicrobial therapy (AMT) used for invasive healthcare-associated infections (HAIs) in paediatrics is scarce. Influence of the final information about the isolated pathogen on the subsequent targeted AMT was investigated in our study.

**Methods:**

Data on 149 children (0–17 years) with blood culture positive HAIs were collected. The causative microbes under investigation were *Staphylococcus aureus*, *Staphylococcus epidermidis*, streptococci, Gram negative rods, and mixed infections were likewise included. For adjusting the antimicrobial regimen, an expert panel evaluated the quality of the targeted AMT and the delay of 72 hours after final microbiology results. AMT was regarded as inappropriate if the pathogen was totally resistant to the used antimicrobials (i) or if the chosen therapy was of not optimal efficacy against the pathogen (ii).

**Results:**

17% of the patients received inappropriate AMT. Half of these infections 13/26 (50%) were treated with an antimicrobial to which the isolate was resistant. Three (3/13, 23%) of these patients received antimicrobials which were totally ineffective according to *in vitro* data. Suboptimal or too broad spectrum AMT was administered to 13/26 (50%) patients. The most common causes of inappropriate use were the use of beta-lactams in oxacillin-resistant *Staphylococcus epidermidis* infections and vancomycin given in oxacillin-sensitive *Staphylococcus aureus* infections.

**Conclusion:**

Approximately 17% of the selected cohort received inappropriate AMT. More attention should be paid to the appropriate use of antimicrobials, and training of prescribers should be urgently provided.

## Introduction

Bloodstream infections (BSIs) are among the most common healthcare-associated infections (HAIs) in pediatrics [1,2,3]. They cause substantial morbidity and mortality, increase health care costs and often result in a prolonged hospital stay [4]. They are caused by a vast variety of pathogens [5,6,7] and when a BSI is suspected, rapid initiation of empirical antimicrobial therapy (AMT) is of high importance. The selection of appropriate therapy is, however, challenging since it should cover the most likely causative pathogens. It has been shown by Welsh *et al*. [7] that selection of suboptimal therapy affects the prognosis of the children.

On the other hand, the use of an excessively broad spectrum antimicrobial therapy can expose the patient to opportunistic infections caused by multiresistant organisms, fungi or *Clostridium difficile*. In addition, multiple co-morbidities of the patients and several side effects of the antibiotics further complicate the choice of appropriate therapies.

Both HAIs and resistant bacteria are becoming more common, and development of new antimicrobials is scarce. Inappropriate use of AMT occurs in different ways such as the prescribing antibiotics with no or suboptimal efficacy against the pathogens. On the other hand, if the blood culture is positive, targeting the AMT should be executed promptly in order to optimize the therapy. In addition, failure of de-escalation of the therapy should be considered inappropriate as well.

The aim of our study was to investigate retrospectively the appropriateness of the prescribed targeted AMT in pediatric patients with healthcare-associated BSIs caused by different pathogens. For that purpose we created five different groups of infections based on different pathogens. The causative microbes were the following: *Staphylococcus aureus*, *Staphylococcus epidermidis*, streptococci, Gram negative rods, and mixed infections. In particular, the study focused on how the blood culture results were taken into account after the microbiological information was obtained, i.e. whether the data influenced the subsequent choice of antimicrobials or not. We allowed the colleagues 72 hours (study period) to respond to the final blood culture results.

## Methods

### Study design and participants

The Hospital for Children and Adolescents, Helsinki University Central Hospital, Finland, is a tertiary-care paediatric centre with 130 beds and approximately 100 annual blood culture positive BSIs. The most common blood isolates have been coagulase negative staphylococci (CONS) covering approx. 50% of all isolates. In order to obtain a more heterologous material for our analysis we decided to create five different groups of blood culture positive infections caused by different pathogens (*Staphylococcus aureus*, *Staphylococcus epidermidis*, streptococci, Gram negative rods, and polymicrobial infections). This was done due to the natural occurrence of the causative bacteria in BSIs. Since Gram positive bacteria are the causative agents in most cases. We likewise aimed at collecting Gram negative bacteria since they are generally more virulent and more complex to treat. The aim was to gather approximately 30 patients/group.

**Fig 1:**
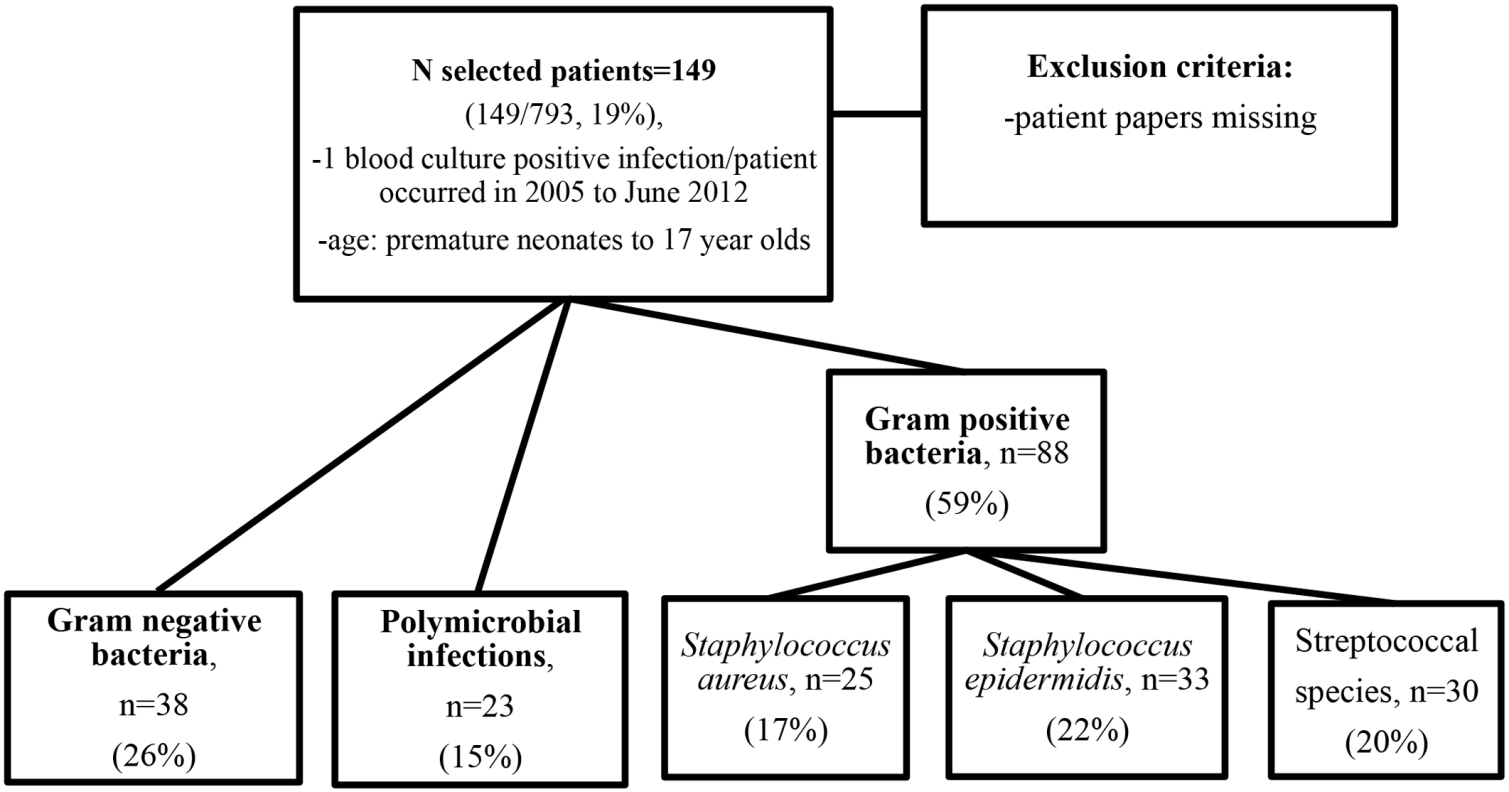
Flow chart of patients selected for the study. The five pathogen groups chosen for the study were: Gram negative bacteria, Polymicrobial infections, *Staphylococcus aureus*, *Staphylococcus epidermidis* and streptococcal species.

Data from patients who suffered from infections caused by *S*. *aureus*, *S*. *epidermidis*, streptococci, Gram negative rods and polymicrobial infections were collected retrospectively in the order of appearance from June 2012 backwards (Fig 1). Due to the fact that *S*. *epidermidis* infections may often be caused by contaminants from the skin, patients with this bacterium were screened carefully into this study in order to include only those patients who had a blood culture positive infection (in most cases: fever and elevated leukocytes were present). Infections caused by fungi and anaerobes were uncommon, and were therefore not analyzed.

The following characteristics were recorded for each patient (if available): age, gender, duration of hospitalization, diagnosis and co-morbidities, weight, height, drug allergies, the use of antimicrobials 7 days prior to the diagnosis, the date of blood sample taken, the date of final blood culture results (i.e. when pathogen was identified and the antibiogramme was given), leukocytes (neutrophils), empirically used antimicrobials, duration of empirical AMT days prior to receiving blood culture results, the antimicrobials selected after the blood culture result. For all antimicrobials used, we recorded: the duration of AMT in days in total, antimicrobial used, dosing, formulation and duration of therapy individual antimicrobial. Our surveillance period was from seven days prior to receiving the positive blood culture to 30 days after the AMT for a healthcare-associated BSI was stopped or to the death of a patient.

### Evaluation of the appropriateness of the targeted antimicrobial therapy

The main focus of the study was the use of antimicrobials during the 72-hour time window immediately after the final data of a positive blood culture was provided by the microbiology laboratory. Thus, the physician(s) in charge were allowed 72 hours to respond to the microbiological data. If necessary changes in AMT were not made during this given time period (of 72 hours), the targeted AMT was considered inappropriate. The 72-hour time window is commonly used when assessing the appropriateness of AMT. The quality of the given targeted AMT for each patient was evaluated retrospectively by an expert panel of three physicians: microbiologist (MV) and two infectious diseases consultants (V-JA and HS). Decision over whether the AMT of a particular patient was inappropriate or not, was a consensus. This study did not evaluate the initial empiric therapy.

When evaluating the quality of therapy for healthcare-associated BSI, the following demographic characteristics were taken into account by the panel: age, co-morbidities, causative pathogen/pathogens, temperature (if the patient had fever) and the level of granulocytes. A leukocyte count of < 1,0 x 10^9^/L was considered as neutropenia.

Inappropriate targeted AMT was divided into two categories: 1) the isolated pathogen was resistant to the selected antimicrobial(s), 2) the isolated pathogen was either treated with an antimicrobial with suboptimal efficacy or the pathogen was treated with too broad spectrum agents, the therapy thus not being optimal. Each patient was categorized into one category solely according to the clinical significance of the inappropriateness of AMT. The analysis used in the study was both quantitative and qualitative.

### Methods and definitions

Hospital-acquired bacteremia was determined according to the classic CDC criteria, where laboratory-confirmed bloodstream infection is LCBI [8]. LCBI is equivalent to the determination of healthcare-associated BSI in our study.

Flow chart of the selected patients is shown in Fig 1.

The study was reviewed and approved by the local Ethical Committee for Women and Children (HUCH, Helsinki University Central Hospital, Finland). Due to the nature of this study (retrospective registry study), no written or verbal consent from the guardians of the children were needed.

This study was a retrospective analysis regarding information found from patient records (paper and electronic). Patient records/information was anonymized and de-identified prior to analysis.

## Results

### Patient profiles and epidemiology

Demographic data of the cohort of 149 patients with different causative bacteria are given in Table 1. 72/149 (48%) patients were both term and preterm neonates (≤ 28 days of age), the age of 24/149 (16%) was between 29 days and 1 year, 39/149 (26%) were between the age of 1 and 12 years and 14/149 (9%) were between the age of 12 and 17 years. The most common malignancy was acute lymphoblastic leukemia and the most common cause of surgery was congenital heart malformation.

**Table 1:** Demographic data of the selected patients with healthcare-associated bloodstream infections (n = 149).

Pathogen group/co-morbidities	Patients/pathogen group	Full term neonates(<28 days)	Premature neonates(born < 37 weeks old)	Patients with malignancies	*Surgical patients	^Other co-morbidities
	no of patients, (%)	no of patients, (%)	no of patients, (%)	no of patients, (%)	no of patients, (%)	no of patients, (%)
***Staphylococcus aureus***	**25 (17)**	3 (12)	6 (24)	5 (20)	8 (32)	3 (12)
***Staphylococcus epidermidis***	**33 (22)**	1 (3)	8 (24)	12 (36)	10 (30)	2 (6)
**Streptococcal species**	**30 (20)**	18 (60)	4 (13)	5 (17)	-	3 (10)
**Gram negative bacteria**	**38 (26)**	8 (21)	14 (37)	3 (8)	9 (24)	4 (11)
**Polymicrobial infections**	**23 (15)**	1 (4)	9 (40)	12 (52)	1 (4)	-
**Total**	**149 (100)**	**31 (21)**	**41 (28)**	**37 (25)**	**28 (19)**	**12 (8)**

*Surgical patients were patients who had undergone surgery during the same hospital admission as they had BSI.

^**^**^Other co-morbidities were defined as patients with co-morbidities that could not be classified to any of the given categories.

Organisms of the streptococcal cohort were the following: *Streptococcus agalactiae* (Group B streptococci, GBS), (18/30, 60%), *Streptococcus viridans* (7/30, 23%), *Streptococcus pyogenes* (3/30, 10%), *Streptococcus pneumoniae* (1/30, 3%) and *Streptococcus salivarus* (1/30, 3%) (Table 1). All of the children infected by GBS were neonates.

The staphylococcal cohorts consisted of patients infected with *Staphylococcus aureus* (n = 25) or *Staphylococcus epidermidis* (n = 33). *S*.*aureus* was most common on surgical patients (8/25, 32%), *S*.*epidermidis* in patients with malignancies (12/33, 36%) (Table 1). No MRSA infections were recorded.

The Gram negative cohort consisted of the following: *Escherichia coli* (19/38, 50%) followed by *Klebsiella pneumoniae* and *oxytoca* (6/38, 16%), *Pseudomonas aeruginosa* (4/38, 11%), *Enterobacter cloacae* (3/38, 8%), *Serratia marcescens* (2/38, 5%), *Citrobacter* (n = 1), *Stenotrophomonas maltophilia* (n = 1), *Sphingomonas paucimobilis* (n = 1), and an unidentified *Enterobacteriaceae* strain (n = 1). Two patients suffered from bacteremia caused by an ESBL strain of *E*.*coli*.

Causative microbes of the polymicrobial group were the following: Gram+ Gram+ (9/23, 39%), Gram+ Gram- (9/23, 39%) and Gram- Gram- (5/23, 22%). In the case of two polymicrobial infections, three different pathogens were isolated form the blood culture.

### Adjusting the empirical use of antimicrobials

The initial empirical antimicrobial therapy of 114 patients (77%) was changed during the study period within three days (72 hours) after receiving the final blood culture results. Empirical AMT was changed in 87% of cases in the polymicrobial infection group (20/23). In the other groups the treatments were changed as follows: *Staphylococcus aureus* 20/25 (80%), *Staphylococcus epidermidis* 26/33 (79%), Gram negative bacteria 27/38 (71%) and streptoccocci 21/30 (70%).

### Inappropriate use of targeted antimicrobials

AMT was considered totally inappropriate in three cases because the pathogen was resistant to all prescribed antimicrobials (Table 2) In one case oxacillin resistant *S*. *epidermidis* (MRSE) was treated with cloxacillin, in another case MRSE was treated with cefuroxime and fluoroquinolone to which the strain was also resistant. One child received cefuroxime monotherapy for *Pseudomonas sp*.

**Table 2:** Patients (n = 3) who were prescribed an entirely inappropriate therapy* due to resistance of the pathogen. Antimicrobial therapy (AMT) given 0 to 72 hours after identification of the pathogen and testing its antimicrobial sensitivity. AMT was given parenterally (IV).

Age	Co-morbidities	Isolate	Resistant to	AMT given
< 12months	left ventricular hypoplasia	*S*. *epidermidis*	oxacillin	cloxacillin*
< 12months	biliary atresia	*S*. *epidermidis*	clindamycin oxacillin levofloxacin	ceftriaxone* ciprofloxacin
< 5 years	transposition of great arteries	*Pseudomonas sp*.	cefuroxime	cefuroxime*

Ten patients received at least one antimicrobial agent to which the pathogen was resistant (Table 3). Five children with MRSE infections were treated with beta-lactams. Two patients with infections caused by *Klebsiella* received vancomycin. One child with an ampicillin-resistant *Citrobacter freundii* infection was treated with ampicillin. One child with a cefuroxime-resistant *Stenotrophomonas* infection was treated with cefuroxime and one child with *E*. *coli* resistant to ciprofloxacin and penicillin was treated with ciprofloxacin and penicillin G.

**Table 3:** Patients (n = 10) who were prescribed an inappropriate therapy* due to resistance of the pathogen to one of the chosen antimicrobials. Antimicrobial therapy (AMT) given 0 to 72 hours after identification of the pathogen and testing its antimicrobial sensitivity. AMT is given parenterally (IV) if not mentioned otherwise.

Isolate	N	Pathogen resistant to	AMT given
*S*. *epidermidis*	1	oxacillin	cefuroxime* netilmycin
*S*. *epidermidis*	1	oxacillin	cefuroxime* clindamycin
*S*. *epidermidis*	1	oxacillin	cefuroxime* metronidazole
*S*. *epidermidis*	1	oxacillin	vancomycin meropenem*
*S*. *epidermidis*	1	oxacillin	teicoplanin meropenem*
*Citrobacter sp*.	1	ampicillin	penicillin G* netilmycin
*Stenotrophomonas maltophilia*	1	cefuroxime	cefuroxime* trimethoprim-sulfamethoxazole (PO)
*E*. *coli*	1	cefuroxime (ESBL) ciprofloxacin	penicillin G* meropemen ciprofloxacin*
*Klebsiella pneumoniae*	1	vancomycin	vancomycin* cefuroxime
*Klebsiella pneumoniae*	1	vancomycin	vancomycin* metronidazole cefuroxime

Out of all 149 cohort patients, 13 (9%) received inappropriate AMT due to suboptimal or too broad spectrum efficacy of the agent (Table 4). The most common cause for not optimal use of AMT was the use of vancomycin in methicillin-sensitive *S*.*aureus* (MSSA) (n = 6) infections and in one methicillin-sensitive *S*. *epidermidis* (MSSE) infection. One MSSA infection was treated with penicillin G (and ceftriaxone) and one MSSA with both intravenous cefuroxime and oral cephalexin. One child with a penicillin-sensitive *Streptococcus viridans* was treated with meropenem, and one child with an *Enterobacter cloacae* infection received cefuroxime monotherapy. Finally, one patient with a mixed infection caused by *E*. *cloacae* and *Enterococcus faecalis* received meropenem–an agent with suboptimal activity against the enterococcus. Another mixed infection caused by *Enterococcus faecalis* and an oxacillin-resistant *Staphylococcus sp* was treated with vancomycin, ampicillin and meropenem. Meropenem use was considered unnecessary.

**Table 4:** Patients (n = 13) who were prescribed a suboptimal or too broad spectrum AMT. The chosen therapy was too broad-spectrum^ or of suboptimal efficacy* or involved concomitant use of antimicrobials of the same class • (eg. cephalosporins) against the pathogen. Antimicrobial therapy (AMT) given 0 to 72 hours after identification of the pathogen and testing its antimicrobial sensitivity. AMT is given parenterally (IV) if not mentioned otherwise.

Isolate	N	Reported as	AMT given
*S*. *aureus*	2	oxacillin S	vancomycin*ceftazidime
*S*. *aureus*	1	oxacillin S	vancomycin* ceftriaxone
*S*. *aureus*	1	oxacillin S	vancomycin* meropenem
*S*. *aureus*	1	oxacillin S	vancomycin* cefuroxime
*S*. *aureus*	1	oxacillin S	vancomycin* netilmycin
*S*. *aureus*	1	oxacillin S	penicillin G* ceftriaxone
*S*. *aureus*	1	oxacillin S	cefuroxime **•** cefalexine • (PO)
*S*. *epidermidis*	1	oxacillin S	vancomycin* netilmycin
*S*. *viridans*	1	ampicillin S	meropenem^
*Enterobacter cloacae* coliform rod	1	ampicillin R	cefuroxime*§ (monotherapy)
*E*. *cloacae*, *Enterococcus faecalis* Streptococcal species	1	ampicillin R ampicillin S	meropenem*§§ (suboptimal enterococcal Rx)
*Enterococcus faecalis* Staphylococcal species	1	ampicillin S oxacillin R	vancomycin meropenem^ ampicillin

^§^ therapy of *E*. *cloacae* with cefuroxime monotherapy not optimal

^§§^ therapy of *E*. *faecalis* with meropenem not optimal

**Fig 2:**
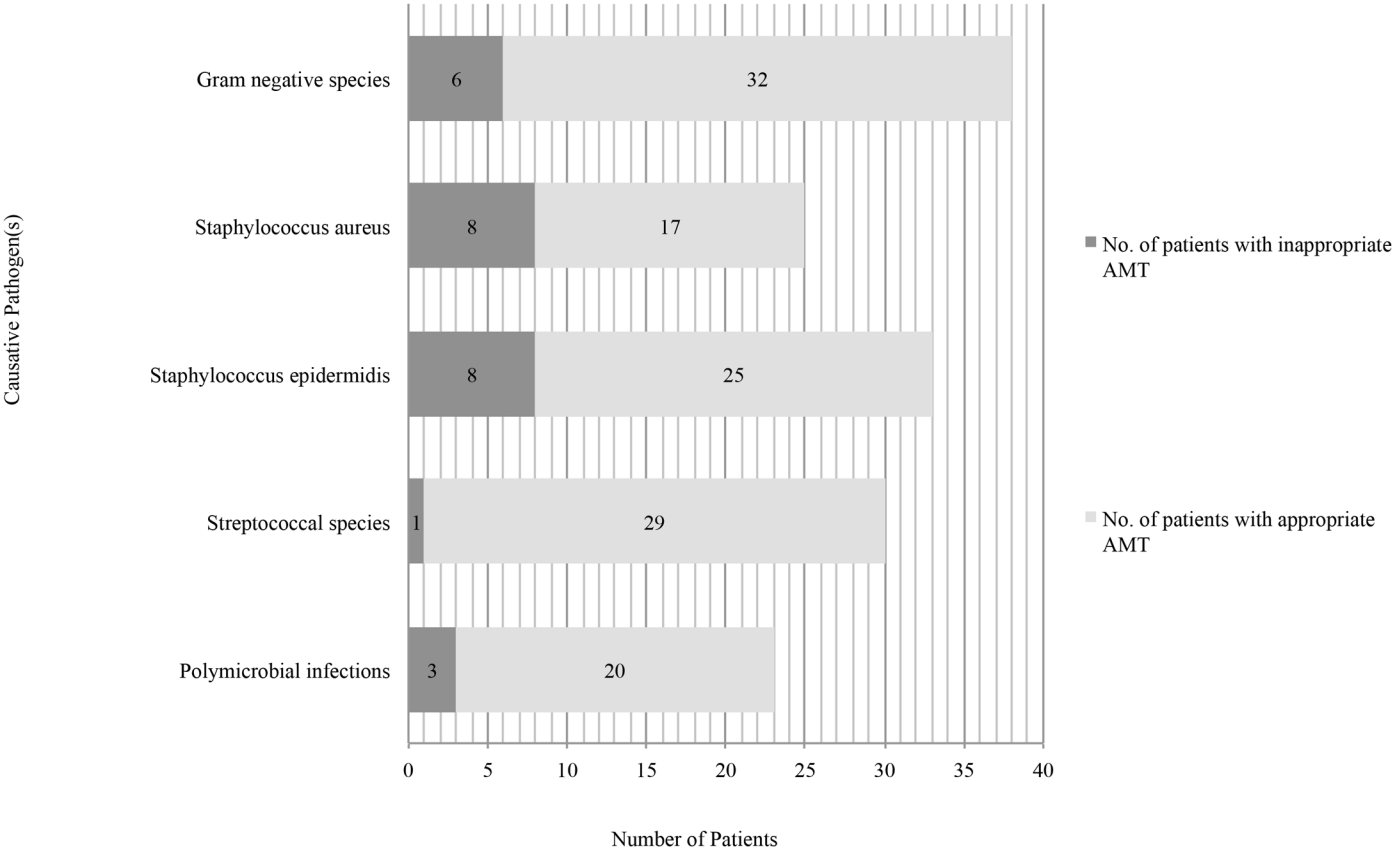
26 patients (26/149, 17%) received inappropriate AMT in 0 to 72 hours after receiving blood culture results.

Inappropriate AMT was found to be most common in the treatment of BSIs caused by *Staphylococcus aureus* (8/25, 32%) (Fig 2). On the contrary, inappropriate AMT was uncommon in infections caused by streptococcal species: only one patient, with ampicillin sensitive *Streptococcus viridans*, received inappropriately meropenem (1/30, 3%).

Lack of de-escalation, too broad spectrum AMT, was a concern in the case of two patients: *S*.*viridans* infection treated with meropenem, and a polymicrobial infection (caused by *Enterococcus faecalis* and staphylococcal species) treated with meropenem (Table 4).

### Outcome of antimicrobial therapy

Mortality rate was low. From the cohort (N = 149 patients), seven patients died (5%). Out of these seven patients, infection was the main cause of death in two cases. Three of the fatal deceased premature neonates (< 1 kg) with BSIs caused by *E*. *coli*, one premature neonate (< 2 kg) with a malignancy had *S*. *epidermidis* BSI, one premature neonate (< 2,5 kg) had a BSI caused by *E*. *cloacae*, one infant 6 months of age with a malignancy had a *S*. *aureus* BSI, one infant 9 months of age with a malignancy had a BSI caused by *S*. *epidermidis* + *S*. *viridans*. Neither of these patients died due to inappropriate AMT. However, the study design did not allow us to demonstrate a causal relationship between the patients that died and inappropriate AMT.

## Discussion

### Summary of key findings

To our knowledge, this is the first study investigating the quality of AMT in children with healthcare-associated BSIs. We focused on one hand on the vigilance of physicians in responding to microbiological results and on the other hand on the appropriateness of the targeted antimicrobial therapy. Our approach of using a selected cohort consisting of different causative pathogens did not allow us to calculate the total magnitude of inappropriate use of antimicrobials of all blood culture positive infections at our hospital. It did, however, give us a general picture of what pathogens were most often targeted with inappropriate AMT.

In all, we discovered that 77% of the patients in the cohort had their empirical AMT changed after receiving the final blood culture results, and 17% of these patients with healthcare-associated BSIs received inappropriate targeted AMT. The microbiological results were frequently either totally (2%) or partly ignored (17%) in the design of the subsequent treatment. Similarly, the choice of antimicrobial agents with suboptimal or too broad spectrum efficacy was not uncommon (9%). To our surprise, inappropriate use of vancomycin in treating MSSA was the most frequent cause of inappropriate use. The second most common misuse was treatment of MRSE infections with beta-lactams. In all, beta-lactams were the most often misused group of antimicrobials.

Inappropriate AMT was not associated to mortality. In general, AMT should have been given to the patients included in this study. All of the patients included had blood culture positive infections with pathogens identified from the blood. In some cases pathogens might have been contaminants with relatively minor virulence, hence it might suggest that AMT was not necessary in all cases.

### Literature and inappropriateness of antimicrobial therapy

Investigating the quality of AMT is a complex topic entailing multiple factors to be considered. The quality of AMT, i.e. appropriate vs. inappropriate therapy, has not been studied extensively. Majority of the studies have focused on infections in adults. These studies have applied different approaches and used a large variety of definitions of appropriate AMT. This diversity makes it difficult to relate our results to those published earlier. The most often used approach has been evaluation of empiric therapy of serious infections [9,10,11]. Some studies have, however, also looked at targeted therapy [12,13,14,15,16,17]. Part of the studies focused especially on verified BSI [9,10,11,16] while others focused on the quality of the AMT in general [12,13,15,17].

Six of the studies used relatively simple definitions of inappropriate AMT [9,10,11,13,15,16]. One study Davey *et al*. [13], defined the AMT inappropriate if the pathogen was resistant to the used antimicrobial agent or initiation of appropriate AMT was delayed. This study concluded that if a patient received appropriate empirical AMT and the therapy was thereafter promptly targeted, the outcome was better than if the empirical AMT had been inappropriate, but had been switched to appropriate targeted therapy. Appropriate targeted AMT is therefore of crucial importance especially if the empirical AMT has been inappropriate. In our study the empirical AMT of 77% of patients was changed after receiving the final blood culture results.

Other studies had used more complex and detailed definitions on the appropriateness of AMT [12,14,17]. Willemsen I. *et al*. used a score system in order to evaluate the appropriateness of the AMT [17]. The score system divided their therapies into five different categories: correct decision, incorrect decision, incorrect choice, incorrect use and data missing. They further speculated that upon evaluating incorrect use, also the following parameters should be taken into account: dosage, timing, administration and duration of therapy. Due to the retrospective nature of our study and the fact that we evaluated solely the choice of antibiotics, we may assume that the frequency of inappropriate use of AMT was even higher than that reported.

Regarding the choice of antimicrobial, excessive use of vancomycin is reported by several studies. The study of Cosgrove SE. *et al*. investigated prospectively clinicians´ antimicrobial prescriptions for different empirical healthcare infections [12]. AMT was criticized in 30% (334/1104) of the cases. In 60/334 (18%) cases unnecessary use of vancomycin was recorded [12]. Patel SJ. *et al*. investigated the use of AMT in neonatal infections [18]. Their main findings were similar to those of ours: the most often misused antimicrobials were vancomycin and carbapenems. The reason for excessive use of vancomycin is not clear. One reason may be that many guidelines of empiric therapy include vancomycin but once the data on the pathogen is exposed, de-escalation is not executed. Increased provider awareness of drug-resistant CONS probably also increases the overall use of vancomycin. Similar findings have been published by Levy ER *et al*. [19].

Two studies investigated whether local or national guidelines or recommendations were followed [15,17]. In the study of Raineri *et al*. a systematic infectious diseases specialist consultation program was implemented in ICU setting and after the implementation, adherence to local guidelines regarding empirical AMT was increased by more than 20% (63% vs. 84%) [15]. Willemsen I. *et al*. also investigated the appropriateness of AMT and found out that during six prevalence studies conducted in 2001 to 2004, the appropriateness of AMT did not vary appreciably. The appropriateness of AMT was evaluated against the local AMT prescription guidelines. Out of 938 patients with AMT, 351 (37%) received inappropriate AMT. Compared with our results, this indicates more inappropriate use of AMT when compared to our results, but it must be taken into account that our study evaluated only the choice of the antimicrobial and targeted AMT instead of empirical AMT.

The proportion of patients receiving inappropriate AMT has varied between studies. The percentage of patients receiving inappropriate AMT varied (20–24%) in empirical AMT [10,11,14] and in targeted AMT (16–37%) [14,15,16]. Due to the small number of studies investigating the appropriateness of AMT, it is unclear how often inappropriate AMT has been used in the treatment of patients with HCA BSIs. The results of our study (17% of inappropriate use of AMT) are, however, in line with the above-mentioned studies.

Appropriate empirical AMT for BSI is known to reduce mortality in empirical sepsis [10] and in enterococcal infections [16]. In addition, Erbay A. *et al*. showed that in bacteremias caused by *Acinetobacter baumanii* infections a 26% reduction in the overall crude mortality rate was achieved with adequate early empirical antimicrobial therapy compared with inadequate therapy [9]. Similarly, Kumar A. *et al*. showed that inappropriate empirical AMT in septic shock was associated with a five-fold increase in mortality [14]. In addition, Raineri E. *et al*. demonstrated in patients with different types of serious infections that appropriate AMT was associated with decreased mortality [15]. In contrast to these studies, Zaragoza R. *et al*. showed that inappropriate AMT was not associated with increased mortality [11]. This finding is in accord with our study where the inappropriate AMT was not associated with increased mortality. A plausible reason for this is that the infections in our hospital were rarely fatal due to the low virulence of the causative pathogens. Hence, most of the patients receiving inappropriate AMT suffered from infections caused by CONS.

### Limitations

Our study involved several limitations. Firstly, our study was retrospective and we were able to collect only data that was recorded. Secondly, the number of patients in our study was relatively small and therefore general assumptions regarding the overall occurrence of true faults are difficult to calculate. Thirdly, the antimicrobials used were evaluated by an expert panel. The classification (appropriate vs. inappropriate) was of course a subjective opinion of the panel. The evaluation was, however, based on microbiological *in vitro* data. Fourthly, the pathogens isolated from the blood cultures may not always represent the sole pathogens causing the infection. This may result in treating children with severe underlying conditions with broad-spectrum drugs (‘just in case’).

### Concluding remarks

Inappropriate therapy of blood-culture positive HAIs was not uncommon. In our cohort unnecessary use of vancomycin in oxacillin-sensitive *Staphylococcus aureus* infections and inadequate use of beta-lactams in oxacillin-resistant *Staphylococcus epidermidis* infections were the most common causes of inappropriate use of antimicrobials.

The co-operation between clinicians, nurses and microbiology laboratory is of vital importance. At present the microbiologist informs the clinician about the blood culture findings. Unfortunately, such service is provided only during office hours. In the future this communication must be improved. Multidisciplinary actions should be enhanced and new forms of multidisciplinary working taken into consideration. Example of a new approach could be the hiring of an infectious diseases pharmacist. This approach has been used in many countries in different settings relating to the rational use of antimicrobials and the impact has been good [20,21,22,23].

The spectrum of pathogens isolated from invasive infections in our hospital was similar to that reported from many other hospitals i.e. staphylococci are the most common causes of infections. During the years 2005–2013, the most frequent isolate was *S*. *epidermidis* (46% of isolates). *S*. *aureus* was isolated from 8% of HAIs. If these infections are in general treated with antibiotics similar to those in our cohort, the absolute number of patients receiving inappropriate antimicrobial agents is much higher than the numbers we have presented here.

Inappropriate use of AMT should not be overlooked. The results of our study were alarming to us, and training of prescribers of antimicrobials by using the presented data has already been initiated. The follow-up of consumption of antimicrobials in defined daily doses at different locations of the Hospital has been initiated. Finally, launching an antimicrobial stewardship program is in progress.

In the future, prospective studies should be carried out in order to further assess the appropriateness of AMT in children with severe HAIs. Regular audits regarding the appropriate use of AMT could be conducted as point prevalence studies.

## References

[1] Burgner D, Dalton D, Hanlon M, Wong M, Kakakios A, Isaacs D. Repeated prevalence surveys of paediatric hospital-acquired infection. J Hosp Infect 1996; 34: 163–170. 8923270 10.1016/s0195-6701(96)90062-6

[2] Frank M, Gur E, Givon-Lavi N, Peled N, Dagan R, Leibovitz E. Nosocomial bloodstream infections in children and adolescents in southern Israel: a 10-year prospective study (1992–2001). Scand J Infect Dis. 2005; 37(3):177–83. 15849049 10.1080/00365540410020956

[3] Balaban I, Tanır G, Metin Timur O, Oz FN, Aydın Teke T, Bayhan GI, et al Nosocomial infections in the general pediatric wards of a hospital in Turkey. Jpn J Infect Dis. 2012 7;65(4):318–21. 22814155

[4] Mühlemann K, Franzini C, Aebi C, Berger C, Nadal D, Stähelin J, etal Prevalence of nosocomial infections in Swiss children’s hospitals. Infect Control Hosp Epidemiol 2004; 25: 765–771. 15484802 10.1086/502474

[5] Sarvikivi E. Lyytikainen O. Vaara M. Saxen H. Nosocomial bloodstream infections in children: an 8-year experience at a tertiary-care hospital in Finland. Clinical Microbiology & Infection. 14(11):1072–5, 2008 11. 19040479 10.1111/j.1469-0691.2008.02079.x

[6] Raymond J, Aujard Y. Nosocomial infections in pediatric patients: a European, multicenter prospective study. European study group. Infect Control Hosp Epidemiol 2000; 21: 260–263. 10782588 10.1086/501755

[7] WelshKerry J, AbbottApril N, LewisEvan M, GardinerJeanelle M, Kruzel, Lewis MC, et al Clinical characteristics, outcomes, and microbiologic features associated with methicillin-resistant Staphylococcus aureus bacteremia in pediatric patients treated with vancomycin. Journal of Clinical Microbiology. 48(3):894–9, 2010 10.1128/JCM.01949-09 20089758 PMC2832419

[8] Garner JS, Jarvis WR, Emori TG, Horan TC, Hughes JM. CDC definitions for nosocomial infections. Am J Infect Control 1988;16:128–40. 2841893 10.1016/0196-6553(88)90053-3

[9] Erbay A, Idil A, Gözel MG, Mumcuoğlu I, Balaban N. Impact of early appropriate antimicrobial therapy on survival in Acinetobacter baumannii bloodstream infections. Int J Antimicrob Agents. 2009 12;34(6):575–9. 10.1016/j.ijantimicag.2009.07.006 19740628

[10] Harbarth S, Garbino J, Pugin J, Romand JA, Lew D, Pittet D. Inappropriate initial antimicrobial therapy and its effect on survival in a clinical trial of immunomodulating therapy for severe sepsis. Am J Med. 2003 11;115(7):529–35. 14599631 10.1016/j.amjmed.2003.07.005

[11] Zaragoza R, Artero A, Camarena JJ, Sancho S, González R, Nogueira JM. The influence of inadequate empirical antimicrobial treatment on patients with bloodstream infections in an intensive care unit. Clin Microbiol Infect. 2003 5;9(5):412–8. 12848754 10.1046/j.1469-0691.2003.00656.x

[12] Cosgrove SE, Patel A, Song X, Miller RE, Speck K, Banowetz A, et al Impact of different methods of feedback to clinicians after postprescription antimicrobial review based on the Centers For Disease Control and Prevention's 12 Steps to Prevent Antimicrobial Resistance Among Hospitalized Adults. Infect Control Hosp Epidemiol. 2007 6;28(6):641–6. 17520534 10.1086/518345

[13] Davey PG, Marwick C. Appropriate vs. inappropriate antimicrobial therapy. Clin Microbiol Infect. 2008 4;14 Suppl 3:15–21. 10.1111/j.1469-0691.2008.01959.x 18318875

[14] Kumar A, Ellis P, Arabi Y, Roberts D, Light B, Parrillo JE, et al Cooperative Antimicrobial Therapy of Septic Shock Database Research Group. Initiation of inappropriate antimicrobial therapy results in a fivefold reduction of survival in human septic shock. Chest. 2009 11;136(5):1237–48. 10.1378/chest.09-0087 19696123

[15] Raineri E, Pan A, Mondello P, Acquarolo A, Candiani A, Crema L. Role of the infectious diseases specialist consultant on the appropriateness of antimicrobial therapy prescription in an intensive care unit. Am J Infect Control. 2008 5;36(4):283–90. 10.1016/j.ajic.2007.06.009 18455049

[16] Suppli M, Aabenhus R, Harboe ZB, Andersen LP, Tvede M, Jensen JU. Mortality in enterococcal bloodstream infections increases with inappropriate antimicrobial therapy. Clin Microbiol Infect. 2011 7;17(7):1078–83. 10.1111/j.1469-0691.2010.03394.x 20946408

[17] Willemsen I, Groenhuijzen A, Bogaers D, Stuurman A, van Keulen P, Kluytmans J. Appropriateness of antimicrobial therapy measured by repeated prevalence surveys. Antimicrob Agents Chemother. 2007 3;51(3):864–7. 17210766 10.1128/AAC.00994-06 PMC1803106

[18] Patel SJ, Oshodi A, Prasad P, Delamora P, Larson E, Zaoutis T, et al Antibiotic use in neonatal intensive care units and adherence with Centers for Disease Control and Prevention 12 Step Campaign to Prevent Antimicrobial Resistance. Pediatr Infect Dis J. 2009 12;28(12):1047–51. 19858773 10.1097/INF.0b013e3181b12484 PMC4526135

[19] Levy ER, Swami S, Dubois SG, Wendt R, Banerjee R. Rates and appropriateness of antimicrobial prescribing at an academic children's hospital, 2007–2010. Infect Control Hosp Epidemiol. 2012 4;33(4):346–53. 10.1086/664761 22418629

[20] Buyle FM, Wallaert M, Beck N, Boelens J, Callens S, Claeys G, et al Implementation of a multidisciplinary infectious diseases team in a tertiary hospital within an Antimicrobial Stewardship Program. Acta Clin Belg. 2014 7 16. 10.1179/2295333714Y.0000000045 25027808

[21] Yu K, Rho J, Morcos M, Nomura J, Kaplan D, Sakamoto K, et al Evaluation of dedicated infectious diseases pharmacists on antimicrobial stewardship teams. Am J Health Syst Pharm. 2014 6 15;71(12):1019–28. 10.2146/ajhp130612 24865759

[22] Bartlett JM, Siola PL. Implementation and first-year results of an antimicrobial stewardship program at a community hospital. Am J Health Syst Pharm. 2014 6 1;71(11):943–9. 10.2146/ajhp130602 24830998

[23] Shah PJ, Bergman SJ, Graham DR, Glenn S. Monitoring of Outpatient Parenteral Antimicrobial Therapy and Implementation of Clinical Pharmacy Services at a Community Hospital Infusion Unit. J Pharm Pract. 2014 8 8. 10.1177/0897190014544786 25107418

